# BACE1 RNAi Restores the Composition of Phosphatidylethanolamine-Derivates Related to Memory Improvement in Aged 3xTg-AD Mice

**DOI:** 10.3389/fncel.2016.00260

**Published:** 2016-11-11

**Authors:** Javier G. Villamil-Ortiz, Alvaro Barrera-Ocampo, Diego Piedrahita, Claudia M. Velásquez-Rodríguez, Julian D. Arias-Londoño, Gloria P. Cardona-Gómez

**Affiliations:** ^1^Cellular and Molecular Neurobiology Area, Group of Neuroscience of Antioquia, Sede de Investigación Universitaria, University of AntioquiaMedellín, Colombia; ^2^Food and Human Nutrition Group, University of AntioquiaMedellín, Colombia; ^3^Department of Systems Engineering, University of AntioquiaMedellín, Colombia

**Keywords:** Alzheimer’s disease, phospholipids, BACE1, RNA interference, hippocampus, cognitive function

## Abstract

β-amyloid (Aβ) is produced by the β-secretase 1 (BACE1)-mediated enzymatic cleavage of the amyloid precursor protein through the amyloidogenic pathway, making BACE1 a therapeutic target against Alzheimer’s disease (AD). Alterations in lipid metabolism are a risk factor for AD by an unknown mechanism. The objective of this study was to determine the effect of RNA interference against BACE1 (shBACEmiR) on the phospholipid profile in hippocampal CA1 area in aged 3xTg-AD mice after 6 and 12 months of treatment compared to aged PS1KI mice. The shBACEmiR treatment induced cognitive function recovery and restored mainly the fatty acid composition of lysophosphatidylethanolamine and etherphosphatidylethanolamine, reduced the cPLA2’s phosphorylation, down-regulated the levels of arachidonic acid and COX2 in the hippocampi of 3xTg-AD mice. Together, our findings suggest, for the first time, that BACE1 silencing restores phospholipids composition which could favor the recovery of cellular homeostasis and cognitive function in the hippocampus of triple transgenic AD mice.

## Introduction

Alzheimer’s disease (AD) is a disabling neurodegenerative disorder that impacts millions of people worldwide and is recognized as the most common form of dementia (Querfurth and LaFerla, 2010). The aggregation of three main neuropathological markers—senile plaques (SP), neurofibrillary tangles (NFTs) and lipid granules—is characteristic of AD. Current evidence suggests that senile plaques trigger the amyloid cascade and that the deposition of β-amyloid (Aβ) is the first step in the development of AD, leading to the formation of SP, NFT, neuronal loss, and finally, clinical dementia In AD, neuronal loss alters the cellular composition and macrostructure of cerebral regions, either moderately, as observed in the prefrontal cortex, or severely, as in the entorhinal cortex and hippocampus. Progressive cell death in these regions is tightly linked to dementia due to the reduction in the number of neurons found in the hippocampus and cerebral cortex of individuals with AD but not asymptomatic subjects (Andrade-Moraes et al., 2013). Accordingly, AD patients show significantly less activation in the hippocampal formation, which is associated with progressive memory loss (Sperling et al., 2003).

Aβ is generated by the enzymatic processing of the amyloid precursor protein (APP). This process involves the cleavage of APP by the β-site APP cleavage enzyme 1 (BACE1) and the γ-secretase complex, which contains presenilin 1 or 2 (PS1 or PS2) as the catalytic subunit to generate the Aβ40 and Aβ42 peptides (Thinakaran and Koo, 2008). BACE1 is a type I transmembrane aspartic protease that is related to pepsins and retroviral proteases. The subcellular localization of BACE1 is within the trans-Golgi network and the endosomal compartment. Although, BACE1 reaches the plasma membrane due to vesicle trafficking, it is quickly recycled. Only a limited amount of APP cleavage by BACE1 takes place at the plasma membrane; the primary BACE1-mediated APP processing occurs in endocytic vesicles where the low pH environment favors the enzymatic activity (Kandalepas and Vassar, 2012).

Cellular lipid composition also regulates the catalytic activity of enzymes such as BACE1, as observed in cholesterol-rich membrane domains known as lipid rafts (Ehehalt et al., 2003). The cholesterol content of these membrane domains also seems to affect the enzymatic activity of BACE1 on APP (Kalvodova et al., 2005). However, although cholesterol is the best-studied brain lipid in AD, many other lipids are involved in the Aβ-lipid regulatory system, and some of these lipids exhibit stronger effects than cholesterol on Aβ production (Di Paolo and Kim, 2011).

The association between an aberrant lipid balance and AD is supported by the fact that the neuronal lipid composition regulates the trafficking and activity of the membrane proteins involved in Aβ production, such as APP, BACE1 and presenilins 1 and 2 (Grimm et al., 2005). It has also been found that high levels of Aβ modulate the activity of enzymes such phospholipases A_2_, C, and D and thus alter membrane homeostasis (Berman et al., 2008; Oliveira and Di Paolo, 2010; Sanchez-Mejia and Mucke, 2010). In addition, several genes related to AD, such as APOE, ABCA7, CLU, and BIN1, are tightly connected to lipid metabolism and cellular membrane dynamics (Hollingworth et al., 2011; Karch and Goate, 2014; Rosenthal and Kamboh, 2014).

Although the link between lipid metabolism and AD was suggested years ago, many aspects of this relationship have remained elusive due to the lack of technology and analytical methodologies to understand the molecular mechanisms of lipid alterations in AD. The advent of lipidomics has allowed researchers to detect and quantify the lipid species and FAs of cerebral structures, thus facilitating the analysis of the lipid classes involved in cell signaling, membrane structure and trafficking. These techniques have been used to establish the lipid compositions of brain tissues from healthy individuals and AD patients (Han et al., 2001; Fabelo et al., 2014), revealing changes in the levels of plasmalogens, such as, ePE, sulfatide, ceramide, galactosylceramide, cholesterol, and alkyl-acylglycerophosphocholine (Han et al., 2001, 2002; Cutler et al., 2004).

Nevertheless, there is also evidence suggesting that some of enzymes involved in AD, can regulate lipid homeostasis. Thus, post-mortem analysis of frontal cortices from AD patients revealed a 47% increased activity of BACE1 compared with samples from cognitively normal individuals. They found a positive significant linear correlation between BACE1 activity and the levels of 4-hydroxynonenal (HNE) and malondialdehyde (MDA) (Borghi et al., 2007). This study hints to the fact BACE1 may have a direct effect on regulating lipid homeostasis, which is also supported by the work of Meakin et al. (2012) who used mice lacking *BACE1* gene to evaluate the effect of this enzyme on the body weight, as well as lipid and glucose metabolism. They found that the knockout animals had decreased production of lipids in the body and showed increased insulin sensitivity compared to their littermate controls, which may be an indicative of high energy expenditure. Furthermore, experiments demonstrated that the absence of BACE1 in brown adipose tissue and skeletal muscle led to increased levels of the uncoupling protein 1, which seems to be involved in protection against oxidative stress and fatty acid handling (Meakin et al., 2012).

In addition, our previous results showed that the silencing of BACE1 produces reduction of tauopathy in old 3xTg-AD mice, and part of the effect was autophagosome lipidation-dependent (Piedrahita et al., 2016). Therefore, supported in those previous evidence about the role of BACE1 in lipid homeostasis; our hypothesis is that BACE1 down-regulation affects the phospholipid composition of hippocampus in an AD model. In this study, we present the lipidomic profiles of hippocampal tissue obtained from a commonly used transgenic mouse model of AD (3xTg-AD) treated with RNA interference (RNAi) against BACE1 for 6 and 12 months and compared them with control groups, using mass spectrometry, we detected and analyzed 12 different lipid classes and subclasses, including phospholipids (PLs), sphingolipids, lysophospholipids, plasmalogens, covering over 402 lipid subspecies.

## Materials and Methods

### RNAi Design

We designed the shRNAi sequences for silencing BACE1 (shBACE1miR). The scrambled RNAi sequences used as control (shSCRmiR) were based on previously published sequences (Chang et al., 2006). These sequences were cloned into human miR-30-based stem-loops by polymerase extension of overlapping DNA oligonucleotides. The following primers were used for polymerase extension to clone the RNAi into the lentiviral shuttle plasmid (pCMV-GIN-ZEO.GFP) for transfection in HEK-293T cells: shBACE1miR forward primer, 5′…CAGAAGGCTCGAGAAGGTATATGCTGTTGACAGTGAGCGCGGACTGCAAGGAGTACAACTATAGTGAAGCCACAGATGTA…3′ and shBACE1miR reverse primer, 5′…CTAAAGTAGCCCCTTGAATTCCGAGGCAGTAGGCATGGACTGCAAGGAGTACAACTATACATCTGTGGCTTCAC…3′; shSCRmiR forward primer, 5′…CAGAAGGCTCGAGAAGGTATATGCTGTTGACTAGCACACATCAGGAAGCGCTCGACAGTGATAGTGAAGCCACAGATGTA…3′, and shSCRmiR reverse primer, 5′…CTAAAGTAGCCCCTTGAATTCCGAGGCAGTAGGCA CCTAGCACACATCAGGAAGCGCTCGACAGTGATACATCTGTGGCTTCAC…3′. The extension products were digested with XhoI and EcoRI for directional cloning into the pCMV-GIN-ZEO.GFP vector (Open Biosystems, Pittsburg, PA, USA). To clone the RNAi vectors for adeno-associated virus (AAV) production, the following primers were used for polymerase extension: shBACE1miR forward primer, 5′…AAAACTCGAGGAGCTCGTGAGCGCTGGACTGCAAGGAGTACAACTCTGTGAAGCCACAGATGGG…3′ and shBACE1miR reverse primer 5′… TTTTGGATCCATTAATAGGCAATGGACTGCAAGGAGTACAACTCCCATCTGTGGCTTCACAG…3′; shSCRmiR forward primer, 5′…AAAACTCGAGTGAGCGCACCATCGAACCGTCAGAGTTACTGTAAAGCCACAGATGGG…3′ and shSCRmiR reverse primer, 5′…AAAAACTAGTAGGCGTACCATCGAACCGTCAGAGTTACCCATCTGTGGCTTTACAG…3′. These extension products were digested with XhoI and SpeI for directional cloning into a U6 expression plasmid that was cut with XhoI and XbaI (Boudreau et al., 2009).

### Viral Particle Production and Neuronal Culture Transduction

The AAV particles were obtained by the large-scale production of heterologous proteins from Sf9 insect cell cultures that were co-infected with recombinant baculovirus derived from the *Autographa californica* nuclear polyhedrosis virus (Urabe et al., 2002). The shBACE1miR expression cassettes—driven by the mouse U6 promoter—were cloned into pAAV.CMV.hrGFP, which contained AAV serotype 2/5 inverted terminal repeats and a CMV-humanized *Renilla* GFP (hrGFP)-simian virus 40 poly (A) reporter cassette (Urabe et al., 2002). The AAV titers were determined using quantitative PCR and/or DNA slot blot analysis. The AAV particles were dialyzed before use.

### Animal Procedures

We used 3xTg-AD mice containing PS1 (M146V), APP (Swe), and tau (P301L) mutations (Oddo et al., 2003), as well as the PS1KI mice containing the PS1 (M146V) mutation, named as “control group,” because PS1KI is the genetic background of the 3xTg-AD mice, do not develop intracellular and extracellular β-Amyloid aggregation and do not exhibit LTP deficits. Furthermore, those mice are viable, fertile, normal in size and do not display any gross physical or behavioral abnormalities (Oddo et al., 2003). Both mice strains were kindly donated by Dr. Frank M LaFerla from the University of California (Irvine, CA, USA). The animals were bred in-house in a specific pathogen-free (SPF) colony at the vivarium at SIU (Sede de Investigación Universitaria, University of Antioquia, Medellin, Colombia), maintained with a 12 h:12 h dark:light cycle, and received food and water *ad libitum*. The animals were handled in accordance with the Colombian standards (Law 84/1989 and resolution 8430/1993) and NIH guidelines for animal welfare and care (Public Law 99-158, November 20, 1985, “Animals in Research”). Specific care was taken to minimize animal suffering and minimize the number of animals used. A total of 42 3xTg-AD mice (6 and 12 months old, 20–30 *g*), 20 PS1KI mice (6 and 12 months old, 20–30 g) were used. 28 males and 14 females mice were aleatorially assigned, using three males and 2–3 females per group. One side of the hippocampi was used for lipidomic analysis and the other for biochemical analysis.

**FIGURE 1:**
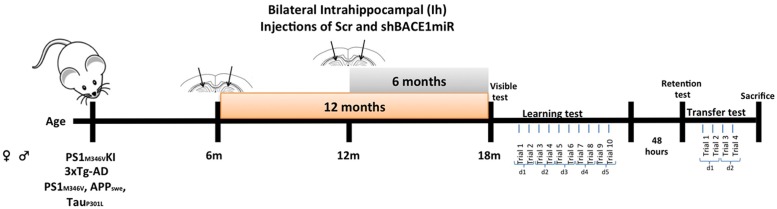
**Experimental design.** AAVshSCRmiR and AAVshBACEmiR were injected in the hippocampus of 6 and 12 months-old PS1KI and 3xTg-AD mice. After, 12 and 6 months post-injection respectively, behavioral analyses using Morris water maze test were realized. First and second days visible test; Learning and memory were evaluate during 5 days (10 trials); after 48 h retention test was realized. Afterward, the transfer test was performed over 2 days (four trials).

The animals were anesthetized (5% ketamine and 2% xylazine, 50:5 dosage mg/kg) and bilaterally injected with 1 μL of AAV2-shSCRmiR (shSCRmiR) or AAV2-shBACE1miR (shBACE1miR) into both hippocampi (bregma coordinates were -1.7 anteroposterior, 0.8 (right) and -0.8 (left) lateral, and 2.5 mm dorsoventral). The injections were performed with a 10-μL syringe (Hamilton, Reno, NV, USA) at 0.1 μL/min, and 10 min elapsed after the infusion before the syringe was withdrawn. The following experimental groups were used:, *Short-term treatment group*, PS1KI and 3xTg-AD mice were injected with shBACE1miR (B12m) or scrambled miR (Scr12m) at an age of 12 months old and evaluated 6 months later; and *Long-term treatment group*, PS1KI and 3xTg-AD mice injected with shBACE1miR (B6m) and scrambled miR (Scr6m) at an age of 6 months and evaluated 1 year after injection (**Figure 1**). The animals were evaluated by the Morris water maze test and later sacrificed for biochemical measurements (**Figure 1**). The hippocampi were dissected, immediately frozen on dry ice and stored at -80°C until use.

### Morris Water Maze Test

Morris water maze testing was performed at 6 and 12 months following the shBACE1miR and shSCRmiR injection in the PS1KI and 3xTg-AD mice. A circular white pool (1-m diameter and 0.5-m height) was filled with water at 22 ± 2°C, and a white escape platform with a diameter of 20 cm was submerged 2 cm below the water surface. This platform was hidden, because the water was colored white with non-toxic opaque paint to facilitate video recording. The test procedure consisted of four stages: (1) Visible: 2 days, two trials per day, 60 s per trial with the platform 2 cm above the water level; (2) Learning: 5 days, two trials per day (10 trials in total), 60 s per trial to find the hidden platform; (3) Retention: 48 h after the last training day (1 day, one trial per day, 60 s per trial without platform); and (4) Transference (changing location of the platform to the opposite side of the pool): a second learning trial of 2 days, two trials per day (four trials in total), 60 s per trial to find the hidden platform (**Figure 1**). The animal behavioral tests were recorded and analyzed individually using View Point software (Lyon, France).

### Lipid Analyses

The total lipids from the mouse hippocampus were extracted according to the FOLCH technique (Folch et al., 1957) using a mixture of 2 mL of chloroform (CHCl_3_) and 1 mL of methanol (MeOH) in a 2:1 (v/v) ratio. Then, 0.005% butylated hydroxytoluene (BHT) was added, and this mixture was used to homogenize the hippocampus. Subsequently, 1 mL of 0.9% NaCl was added, and the mixture was centrifuged at 3000 rpm for 3 min. The organic layer (lower layer) was removed and transferred to a new glass tube. This procedure was performed in an oxygen-free using enriched nitrogen environment to avoid lipid oxidation processes and was repeated three times. The solvents were evaporated, and the extract was lyophilized to remove the excess of humidity. Finally, the lipid composition was analyzed by mass spectrometry.

### Mass Spectrometry

An automated ESI-MS/MS approach was used and data acquisition and analysis carried out at the Kansas Lipidomics Research Center using an API 4000^TM^ and Q-TRAP (4000Qtrap) detection system as described previously (Sparkes et al., 2010; Zhou et al., 2011). This protocol allowed the detection and quantification of low concentrations of the polar lipid compounds. The molecules were determined by the mass/charge ratios, which were compared with the respective internal standard to determine which species of lipids were present in the evaluated extract: 0.66 nmol di14:0-PC, 0.66 nmol di24:1-PC, 0.66 nmol 13:0-lysoPC, 0.66 nmol 19:0-lysoPC, 0.36 nmol di14:0-PE, 0.36 nmol di24:1-PE, 0.36 nmol 14:0-lysoPE, 0.36 nmol 18:0-lysoPE, 0.36 nmol 14:0-lysoPG, 0.36 nmol 18:0- lysoPG, 0.36 nmol di14:0-PA, 0.36 nmol di20:0(phytanoyl)-PA, 0.24 nmol di14:0-PS, 0.24 nmol di20:0 (phytanoyl)-PS, 0.20 nmol 16:0-18:0-PI, 0.16 nmol di18:0-PI, and 1 nmol 15:0 fatty acid. The system detected a total of 12 different lipid species and their respective sub-species, which were identified by the number of carbons and degree of unsaturation of the chain. Lipid concentration was normalized by molar concentration across all species for each sample, and the final data are presented as mean mol%.

### Western Blotting Analysis

The animals were sacrificed, and the cerebral cortices were dissected, immediately frozen in liquid nitrogen, and stored at -80°C prior to use. The samples were lysed in 10 mM Tris (pH 7.4), 100 mM NaCl, 1 mM EDTA, 1 mM EGTA, 10% glycerol, 1% NP40, 1 nM orthovanadate, 5 mM NaF, 1 mM phenylmethylsulfonyl fluoride, and a protease inhibitor cocktail (Sigma-Aldrich) (Cardona-Gomez et al., 2004). The lysates (containing approximately 40 μg of proteins, quantified using the Bradford method) were loaded onto 8 and 10% polyacrilamide gels and transferred onto nitrocellulose membranes (GE Healthcare) at 250 mA for 2 h using an electrophoretic transfer system. The membranes were incubated overnight at 4°C in anti phospho-cPLA2 (Ser505) (rabbit polyclonal. 1:1000; Cell Signaling), anti-COX2 (rabbit polyclonal. 1:500; abcam), and mouse anti-βIII tubulin (1:5000; Promega). IRDye 800CW goat anti-rabbit (LI-COR; 1:5000) were used as the secondary probes. The blots were developed using the Odyssey Infrared Imaging System. To minimize inter-assay variation, samples from all of the experimental groups were processed in parallel.

### Statistical Analysis

In the behavioral test were used 10–15 animals/group. The escape latency during the hidden platform training sessions and transference tests were determined using repeated-measures ANOVA. The latency and the number of platform crosses in the hidden platform trials and in the probe trials were analyzed by one-way ANOVA, also ANOVA per day followed by Fisher’s *post hoc* test were realized. The analyses were performed using SPSS 18.0 software (Chicago, IL, USA). The values were expressed as the mean ± SEM. The results were considered to be significant at ^∗^*p* < 0.05, and ^∗∗^*p* < 0.01. All of the sample groups were processed in parallel to reduce inter-assay variation.

The lipid levels for each sample were calculated by summing the total number of moles of all lipid species measured and then normalizing that total to mol%. Comparisons between groups were assessed either by one-way ANOVA, followed by the Tukey *post hoc* test or the Kruskal–Wallis test, depending on the homoscedasticity and normality of the experimental data. Multivariate statistics were performed using principal component analysis (PCA) and a partial least squares-discriminant analysis (PLS-DA) (Barker and Rayens, 2003). PLS-DA was included because it is particularly suitable for the analysis of datasets with a small number of samples and a large number of variables. The PLS-DA analysis was carried out using the routines described in (Ballabio and Consonni, 2013). For both techniques, an index representing the importance of the variables according to the first components was estimated. PCA used an index (denoted as ρ) that considers the entries of the principal directions and the variance of each component to determine the weight that every lipid species has in the estimation of the principal directions (Jolliffe, 2002). In contrast, for PLS-DA, the index used is called the variable importance in projection (VIP) (Mehmood et al., 2012), which analogously to ρ, determines the importance of each variable that is reflected by each component and also considers the variance explained by each PLS component (for a detailed description of VIP, please refer to Mehmood and the references therein). The confidence ellipsoids per group and treatment are also included. The data from the univariate and bivariate statistics are expressed as the mean ± the standard error of the mean. The statistical significance is indicated in the figures and tables.

## Results

### BACE1 Gene Silencing Prevents Learning and Memory Impairments in 3xTg-AD Mice after 6 and 12 months of Treatment

**FIGURE 2:**
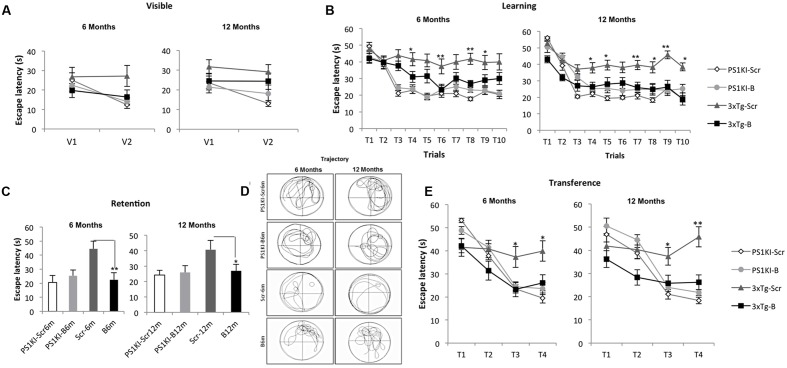
**BACE1miR prevents cognitive dysfunction in 3xTg-AD mice.** The learning and memory task performance of the PS1KI and 3xTg-AD mice were evaluated by the Morris water maze test after 6 and 12 months of treatment with shBACE1miR and compared with the respective animal controls. **(A)** Visible test, **(B)** Learning test (first position of the platform), **(C**,**D)** Retention and representative trajectory images of the animals during the retention test, and **(E)** Transference test (second position of the platform), The data are expressed as the group mean ± SEM. ^∗^*p* < 0.05, ^∗∗^*p* < 0.001; *n* = 12 to 10–15 animals/group.

During the visible platform test, the shBACE1miR-treated 3xTg-AD mice did not show differences compared to the controls after either 6 or 12 months of treatment (**Figure 2A**). However, the learning task performance of the shBACE1miR-treated mice at 6 and 12 months post-injection was better than that of the corresponding shSCRmiR-treated 3xTg-AD control mice (**Figure 2B**). These observations supported a memory improvement because the shBACE1miR-treated mice spent less time finding the hidden platform and showed preferences in the trajectory for the quadrant of the platform (**Figures 2C,D**) at both post-injection time points. Additionally, reversal learning skills were improved in the 3xTgAD mice treated with shBACE1miR, which spent less time looking for the second platform location in the transfer test than the shSCRmiR-treated 3xTgAD mice (**Figure 2E**). PS1KI mice treated with shBACE1miR did not show changes respect to the untreated control in both evaluated time lines (**Figure 2**).

### The Phospholipid Profile of the Hippocampus Is Altered in 3xTg-AD Mice

To determine whether the transduction with shBACE1miR or the scrambled version induced lipid changes in the 3xTg-AD mice, we characterized the hippocampal phospholipid composition of the transgenic animals at two different post-injection time points (6 and 12 months). These profiles were compared with shBACE1miR or the scrambled treated PS1KI mice, which were evaluated at the same post-injection times. At a glance, the analysis revealed two types of variations in the phospholipid contents of the 3xTg-AD mouse hippocampus. The first set of changes seems to be related to the pathological condition, while the second type is associated with the shBACE1miR treatment.

**FIGURE 3:**
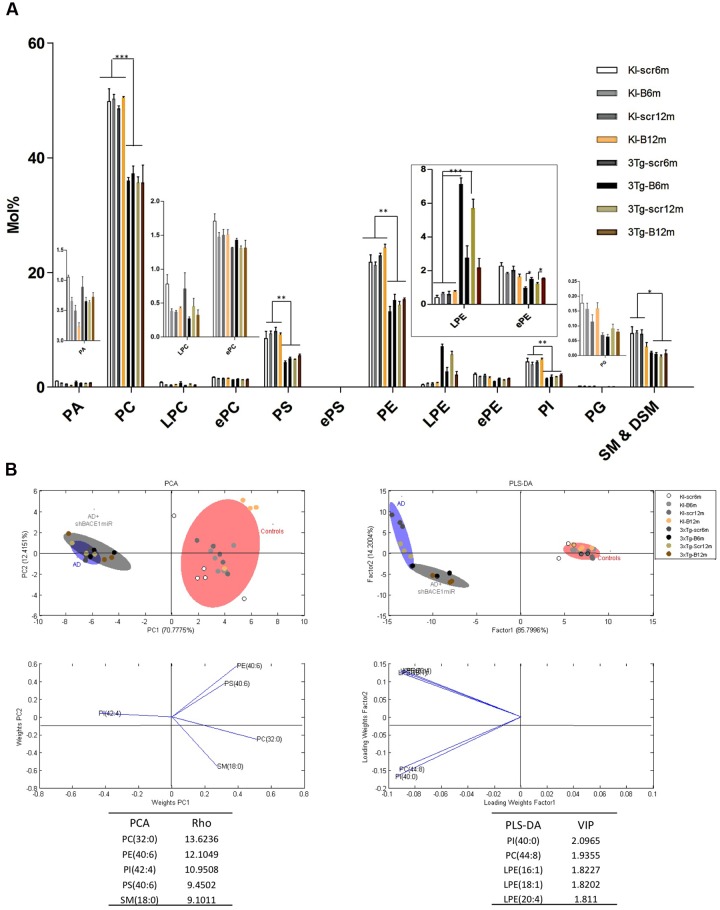
**Lipid composition of the hippocampus from 3xTg-AD and control mice, grouped according to the time of treatment.** Various lipid changes can be seen in the hippocampal tissue. **(A)** The lipid class profiles are expressed as %mol composition. All lipid species were measured (means), and the error bars represent the SEM. The data for the 3xTg-AD mice were significantly different from the control groups (^∗^*p* < 0.05, ^∗∗^*p* < 0.01; ^∗∗∗^*p* < 0.001 ANOVA followed by the Tukey *post hoc* test or Kruskal–Wallis test). **(B)** Multivariate analyses of the lipid profiles from the hippocampus. PCA, principal component analyses for the lipid classes; PLS-DA, partial least squares analysis to discriminate between the lipid classes. The left panels illustrate the factor loadings for PC1 and PC2, with the indices of variance explained for each component. The right panels show the factor score plots for PLS-DA. The variables in the analyses are the controls, AD (3xTg-AD without treatment) and AD+shBACE1-miR (3xTg-AD with 6 and 12 months of treatment). PA, phosphatidic acid; PC, phosphatidylcholine; LPC, lysophosphatidylcholine; ePC etherphosphatidylcholine; PS, phosphatidylserine; ePS, etherphosphatidylserine; PE, phosphatidylethanolamine; LPE, lysophosphatidylethanolamine; ePE, etherphosphatidylethanolamine, PI, phosphatidylinositol; PG, phosphatidylglycerol; SM, sphingomyelin. *n* = 3–5 per group.

The lipid profiling of the all control groups PS1KI and 3xTg-AD mice hippocampus shows that the lipidome is primarily composed of high abundance glycerophospholipids, such as PC (47.5 and 47.2%), PS (5.1 and 5.8%), PE (18.6 and 20.0%), and PI (1.9 and 1.8%); sphingolipids, such as SM-DSM (8.0 and 8.3%); low abundance glycerophospholipids, such as PA (0.3 and 0.4%) and PG (0.07 and 0.6%); lysophospholipids, such as LPC (0.2 and 0.4%) and LPE (0.9 and 1.0%); and etherphospholids, such as ePC (1.4 and 1.6%), ePS (0.02 and 0.11%), and ePE (1.4 and 1.6%) (**Figure 3A**).

The treatment of 3xTg-AD mice with the shBACE1miR or shSCRmiR for 6 or 12 months resulted in distinctive lipid profiles compared with the control animals (**Figure 3A**). The disease-associated changes in the phospholipid composition involve an overall decrease in PC (control vs. AD mice, *p* < 0.001), PS (control vs. AD mice, *p* < 0.01) PE (control vs. AD mice, *p* < 0.01), PI (control vs. AD mice, *p* < 0.01), and SM-DSM (control vs. AD mice, *p* < 0.05) (**Figure 3A**). In contrast, the lipid profile obtained from the shBACE1miR-treated 3xTg-AD mice showed that the treatment restored the LPE (*p* < 0.001) and ePE (*p* < 0.05) content to basal levels (**Figures 3A,B**), suggesting a regulatory role for BACE1 in the biosynthesis of these particular plasmalogens.

The results of the PCA analyses from the detected lipids indicate that nearly 82% of the total variance may be explained by the first two principal components (PC1 and PC2) (**Figure 3B**). The most relevant variables for these two components were related to the PC subclasses 32:0 and PE subclasses 40:6, which demonstrated higher ρ indexes (13.6236 and 12.1049 respectively) (**Figure 3B**). PC 32:0 is composed of the saturated FA Palmitic acid (**Figure 3B**). In contrast, PE 40:6 is composed of the polyunsaturated FAs DHA and saturated FAs stearic acid (**Figure 3B**). The PLS-DA analyses indicate that nearly 86% of the total variance may be explained by factor 1. In this case, the most relevant feature according to VIP are PI 40:00 (VIP: 2.0965) and PC 44:8 (VIP = 1.9355). Moreover, three of the first five most relevant features according to VIP belong to the LPE species (**Figure 3B**). This LPE subclass is composed of the monounsaturated FA such as Palmitoleic (16:1), Oleic (18:1) and polyunsaturated AA acids, as arachidonic (20:4). Both the PCA and PLS-DA analyses indicate that the control group is located in a different quadrant of the plane compared with the AD and treated AD animals, which are in the right quadrant of the graphic (**Figure 3B**). In addition, the AD and treated AD groups also show a different distribution pattern, indicating that BACE1 silencing modifies the course of certain PLs in the disease (**Figure 3B**).

### shBACE1miR Restores the Basal Levels of LPE and ePE of 3xTg-AD at 6 and 12 months Post-injection

**FIGURE 4:**
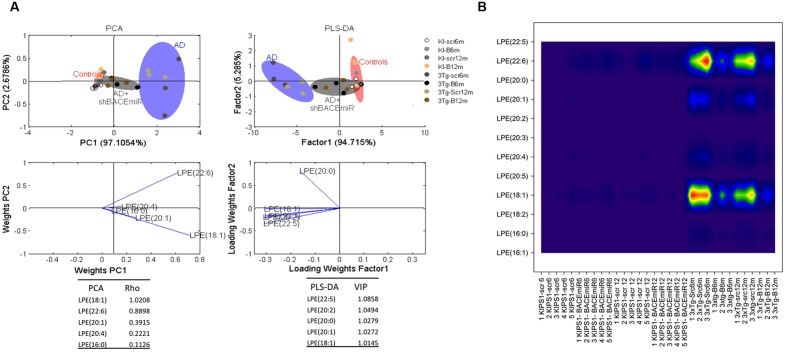
**shBACE1miR restores the basal levels of LPE at 6 and 12 months post-injection.** **(A)** PCA, Principal component analyses for the LPE subclasses and PLS-DA, Partial least squares method to discriminate between the LPE subclasses. The left panels illustrate the factor loadings for PC1 and PC2, with the indices of variance explained for each component. The right panels show the factor score plots for PLS-DA. The groups in the analyses are controls, AD (3xTg-AD without treatment) and AD+shBACE1-miR (3xTg-AD with 6 and 12 months of treatment). **(B)** Contour plots of the more influential subclasses of LPE (variables) in the discriminant analyses for each evaluated variable; all LPE and ePE subclasses were measured (means), and the error bars represent the SEM. *n* = 3–5 per group.

We demonstrated that shBACE1miR has a regulatory effect on the lipid profile in two specific lipid classes: LPE and ePE. The LPE levels increased (*p* < 0.001) in the AD groups compared with the AD mice treated with the shBACE1miR and the control groups (**Figures 3A and 4A**). The PCA analysis showed that the AD+shBACE1miR group had a displacement to the left quadrant close to the control group, while the AD mice occupy a different region that is shifted to the right (**Figure 4A**). This result indicates that nearly 97% of the total variance may be explained by the first two principal components (PC1 and PC2), which is LPE 18:1 according to ρ = 1.0208. However, three of the first five most relevant features according to VIP belong to unsaturated lipid species (22:6, 20:1, and 20:4) (**Figure 4**). In addition, these observations were confirmed by counter plot analyses, where the LPE content was increased in the AD groups (3xTg-Scr6m and 3xTg-Scr12m) and restored to the basal levels in the shBACE1miR-treated AD mice at 6 and 12 months post-injection (**Figure 4B**). The PLS-DA analysis also revealed that the AD+shBACE1miR group on the graph had a displacement from AD to control group; however, the AD group exhibited a different distribution. The subclasses with major importance in data distribution, based on higher ρ index, were LPE 22:5 (docosapentaenoic acid) (1.0858) and LPE 20:2 (Eicosadienoic Acid) (1.0494) (**Figure 4A**).

**FIGURE 5:**
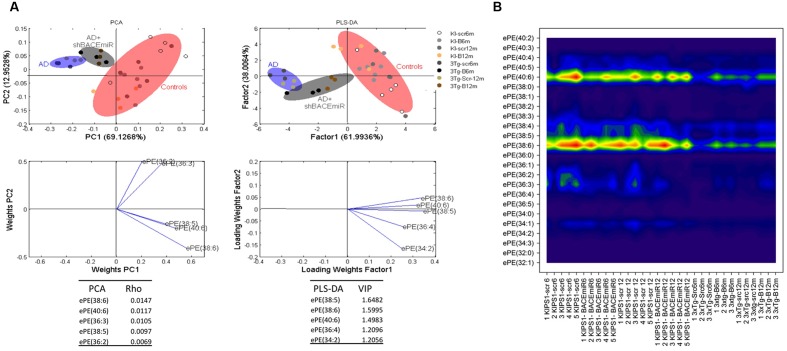
**shBACE1miR recovers the basal levels of ePE at 6 and 12 months post-injection.** **(A)** PCA, principal component analyses for the ePE subclasses; PLS-DA, partial least squares method to discriminate between the ePE subclasses. The left panels illustrate the factor loadings for PC1 and PC2, with the indices of variance explained for each component. The right panels show the factor score plots for PLS-DA. The groups in the analyses are controls, AD (3xTg-AD without treatment) and AD+shBACE1-miR (3xTg-AD with 6 and 12 months of treatment). **(B)** Contour plots of the more influential subclasses of ePE (variables) in the discriminant analyses for each evaluated variable; all ePE subclasses were measured (means), and the error bars represent the SEM. *n* = 3–5 per group.

In addition, ePE also seems to be affected by the treatment because the levels of this plasmalogen decreased (*p* < 0.05) in AD compared with the AD+shBACE1miR and control groups (**Figure 3A**). The PCA analysis confirmed this result and showed that all experimental groups occupy different areas in the quadrants, i.e., ePE species were able to differentiate among the experimental groups. The most relevant molecular species, such as ePE 40:6 (18:0 stearic/22:6 DHA) and 38:6 (16:0 palmitic/22:6 DHA), had a higher ρ index (0,01470 and 1,1170, respectively) (**Figure 5A**). Interestingly, the contour graphic showed changes in the same ePE subclasses and showed a decrease in AD groups, although their levels were recovered in the AD+shBACE1miR groups at 6 and 12 months post-treatment (**Figure 5B**). The recovery of the basal levels may represent a modulatory effect of the shBACE1miR on the phospholipid profile in the 3xTg-AD mice. The PLS-DA analysis also demonstrated different distribution patterns for all groups on the plot. The subclasses with major importance in the data distribution based on a high VIP index were ePE 38:6 (16:0 palmitic acid/22:6 DHA) (1.5995), ePE 40:6 (22:6 DHA/18:0 stearic) (1.4983) and (**Figure 5A**).

**FIGURE 6:**
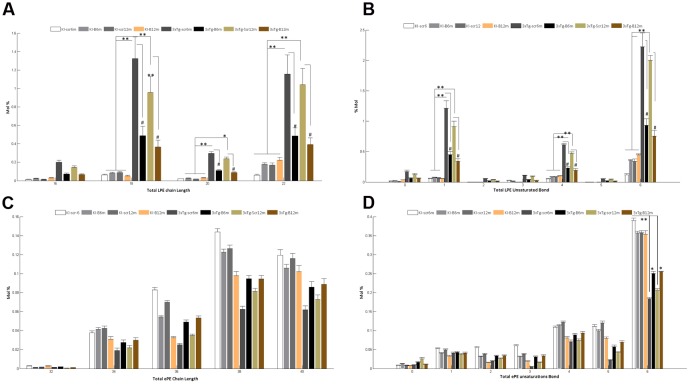
**Altered fatty acid composition of LPE and ePE in the hippocampal region from the 3xTg-AD mouse models and recovery by BACE1miR.** Independent analyses of changes in lysophospholipid (LPE) and plasmalogen (ePE) chain lengths equal to the total number of carbon atoms in the fatty acid moieties **(A,C)**, and the LPE and ePE saturation equal to the total number of double bonds in the fatty acid moieties **(B,D)**. The data for the 3xTg-AD mice were significantly different from those of the control groups (^∗^*p* < 0.05, ^∗∗^*p* < 0.01; ANOVA followed by the Tukey *post hoc* test or Kruskal–Wallis test). #*p* < 0.05, ANOVA with Tukey’s tests compared between 3xTg-AD groups. Data are expressed as Mol %. *n* = 3–5 per group.

A detailed analysis of the LPE’s FA composition revealed the presence of large chain fatty acyls (16C to 22C) with either monounsaturated or polyunsaturated FAs bearing 1, 4, or 6 bonds. Interestingly, the shBACE1miR treatment modified the carbon-chain length and the degree of unsaturation of the LPE subspecies. shBACE1miR seemed to restore the basal levels of long fatty acyls chains, such as 16C, 18C, 20C and 22C, which were altered in the untreated AD groups (3xTg-Scr6m and 3xTg-Scr12m) (**Figure 6A**). The same effect was also found for the degree of unsaturation, particularly in polyunsaturated FAs with 1, 4 or 6 bonds, compared with the control groups (**Figure 6B**). In contrast, the FA composition analysis of ePE showed that this plasmalogen was formed by very long fatty acyls chains (32C to 40C), whose levels did not show statistically significant differences among the experimental groups (**Figure 6C**). In contrast, the analysis of the FA unsaturation number revealed changes in ePE FAs with six bonds (**Figure 6D**). It was evident that the shBACE1miR treatment (3xTg-B6m and 3xTg-B12m) partially restored this type of unsaturation in the ePE FAs to that observed in all control groups (**Figure 6D**).

### BACE1 Silencing Reduces Active cPLA2, ARA, and COX2 Levels

**FIGURE 7:**
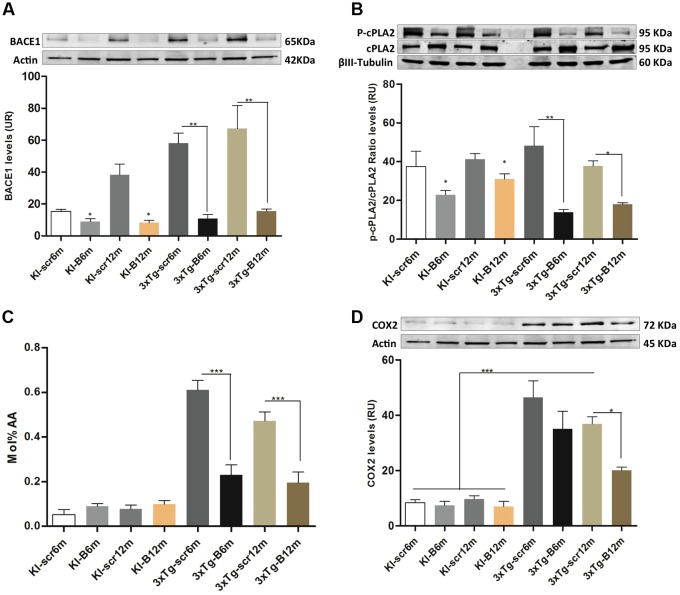
**shBACEmiR decreases the levels of phosphorylated cPLA2, ARA and COX2 levels in the hippocampus of old 3xTg-AD mice’s.** **(A)** Representative bands from BACE1 protein levels and **(B)** p-cPLA2 levels at long and short-term shBACE1miR’s post-treatment (6 and 12 months) by Western blotting. **(C)** Arachidonic acid composition levels in LPE lipid. Results are expressed as Mol % of total fatty acids. **(D)** Representative immunoblot of COX2 protein levels. Results are expressed as relative units (RU) and. Data are shown as mean ± SEM. For 3xTg-AD, results were significantly different from the controls group (^∗^*p* < 0.05, ^∗∗^*p* < 0.01, ^∗∗∗^*p* < 0.001; ANOVA followed by Tukey *post hoc* test). *n* = 3–5 per group.

The LPE and ePE level changes suggested a potential implication of a PLA2 isoform (Dennis et al., 2011), to understand this effect we evaluated the phosphorylation of cPLA, AA levels and COX2 protein levels in all experimental groups, after confirming the decreased BACE1 protein levels (*p* < 0.01) in the hippocampi from shBACE1miR treated mice respect to those treated with shSCRmiR (**Figure 7A**). BACE1 protein levels had a tendency to increase under PS1KI condition, but without significant effect, as reported Giliberto et al. (2009). cPLA2 is the main enzyme involved in the production of both lipids species (Makide et al., 2009), inducing AA release, which trigger proinflammatory signaling through the cyclooxygenases, as COX2 (Lin et al., 1993; Kuwata et al., 1998). Our findings evidenced that the levels of phosphorylated cPLA2 in ser505 were decreased at 6 and 12 months post-injection in the shBACEmiR groups compared with Scr (*p* < 0.01) and PS1KI controls group (*p* < 0.05) (**Figure 7B**). The AA levels were increased in AD (3xTg-Scr6 and Scr12m) groups, how has been previously described (Sanchez-Mejia and Mucke, 2010), while shBACE1miR6 and shBACE1miR12 decreased to control levels (*p* < 0.001) (**Figure 7C**). Finally, we found that COX2 levels increased significantly in all the 3xTg-AD groups; however, this decreased only at the long-term shBACE1miR- treatment (12 m) (**Figure 7D**). Taken together, these results suggest that the BACE1 silencing reduces the composition of AA in LPE at 6 and 12 months of treatment, with a clear repercussion on anti-inflammatory response at 12 months by decrease of cPLA2 and COX2 levels in the hippocampus from 3xTg-AD mice.

## Discussion

Our findings suggest by the first time that the long-term silencing of BACE1 prevents the learning and memory impairment in 3xTg-AD mice, by restoration of the composition of PE derivates, as LPE and ePE, and the subsequent blocking of the pro-inflammatory activation of cPLA2/AA/COX- 2 pathway in the hippocampus. In agreement with our previous studies, this BACE1 knock-down’s effect could be also related to the reduction of the hyperphosphorylation of tau and recovery of PE-dependent autophagosome formation (Piedrahita et al., 2016); since those processes are progressively affected in the 3xTg-AD mice at 12 and 18 months old (Villamil-Ortiz and Cardona-Gomez, 2015).

Recently, we validated the silencing of BACE1 in wild type and triple transgenic AD mice, where shBACE1miR treatment induced neuroprotection by reduction of CTF (carboxy-terminal fragment) and 1–42 Aβ levels. In addition, hyperphosphorylated tau was reduced through the regulation of some autophagy actors, which were blocked by PE lipidation inhibitor [3 Methyl-Adenine (3MA)] (Piedrahita et al., 2016). Therefore, in the present study, we performed a deep lipid profile analysis of the hippocampal region of 3xTg-AD mice to evaluate the effect of BACE1 silencing in this AD model, whose treatment improved the spatial learning and memory skills at 6 and 12 months post-injection. BACE1 inhibition has several substrates involved in neurotransmission, excitability and synapses, as neuroregulin 1, APP, between others (Munro et al., 2016), and its inhibition could be adverse (Vassar, 2014; Ohno, 2016). However, a reduction of BACE1 protein under a pathological over-expressed BACE1 activity condition, could suggest an important benefit in neuroprotection, remyelination and plasticity (Farah et al., 2011; Piedrahita et al., 2016), supported by the recovery of the cognitive function in the shBACE1miR treated 3xTg-AD mice. Also, BACE1’s silencing did not affect the performance of PS1KI mice and it had a lipid profile very similar than C57BL6 mice (**Supplementary Figure S1**).

We detected 12 lipid classes that covered over 402 lipid subclasses, observing changes in 6 PL classes in the hippocampus of the 3xTg-AD mice compared to the control groups. Interestingly, we observed that treatment with shBACE1miR modifies the levels of LPE and ePE, restoring the amounts of these plasmalogens to the basal levels found in the control animals, which may be associated with tissue homeostasis and cognitive function recovery. In addition, there were also changes in the individual lipid species of different subclasses, suggesting that the FA carbon chain and unsaturation number may play an important role in AD pathogenesis. These results suggest that the numerous changes that occurred in the PLs could be used as potential molecular target that facilitate understanding the biochemical mechanism involved in the neurodegenerative process in AD.

Alterations in membrane lipids are widely described in brains from AD and several neurodegenerative disorders (Sagin and Sozmen, 2008). A tight relationship has been established between lipid metabolism and lipotoxicity in neurons (Du et al., 2009). Trafficking and secretase activity of the key membrane-bound proteins controlling Aβ levels, including APP, BACE1, and presenilins, are regulated by membrane levels of cholesterol and SM. According to early studies, a general decline of SM content in AD brains has been described (Cunnane et al., 2012), which was confirmed by decreased levels of SM in the 3xTgAD mice groups in our data. Also, different studies have reported decreased brain PL levels in neurodegenerative diseases, mainly PI (Prasad et al., 1998; Berman et al., 2008; Whiley et al., 2014); PC (Whiley et al., 2014) PE and its derivatives, such as LPE and ePE (Ginsberg et al., 1993, 1995; Nesic et al., 2012); and PS (Shea, 1997), seeming to have multifactorial origins, including the hyperactivation of phospholipases, peroxisomal dysfunction and irregular FA composition of PLs. Our observations confirm those results, where alterations in PL metabolism were close related to AD condition, whose aberrant metabolism could be reflected in the animal’s behavior alteration.

In the present study, we observed decreased levels of PC in all AD groups compared with the control groups, specially the subspecies 34:1 and 32:0. The alterations in the PC levels have been described as a deregulation in the diacylglycerol cholinotransferase-mediated biosynthesis and turnover of PC from DAG, which affects neurite and axonal outgrowth and synaptic plasticity by DAG accumulation and low levels of PC (Araki and Wurtman, 1997; Mateos et al., 2010; Gaudin et al., 2012). However, the biological activity depends on the type of FA linked to the molecular structure. Previous works reported that these PCs are decreased in micro-extracted senile plaques from the post-mortem AD brain, and it could be linked to the roles of PLA_2_ and PLD_1_ in Aβ activation (Gaudin et al., 2012; Whiley et al., 2014). Additionally, these species are composed of monounsaturated omega-9 oleic acid (18:1 *n*-9) and saturated palmitic acid (16:0), and this composition of membrane lipids influences their biophysical properties, including fluidity, permeability and charge (Finkelstein et al., 2014). The loss of oleic acid has been reported to favor BACE1 activity and increase the Aβ 1-42/Aβ 1-40 ratio, whereas dietary oleic acid supplements increased the Aβ 1-40/Aβ 1-42 ratio and reduced the levels of BACE and presenilin, as well as the number of amyloid plaques in AD brains (Amtul et al., 2011). Interestingly, our results confirm the aberrant biosynthesis of PC and the imbalance between the levels of saturated/unsaturated FAs affecting to the structure of PC could favor the abnormal destabilization of membrane in AD brains.

Also, in our work, we found decreased PS and PE levels in the hippocampus from all AD’s experimental groups. Interestingly, we observed that the main decreased FA in both species was 40:6, which is composed of 22:6 (DHA) and 18:0 (stearic acid) chains. DHA is the best substrate for PS biosynthesis, and a decrease in this polyunsaturated acid has been related to cognitive impairment (Grimm et al., 2013). Reduction in the DHA levels of hippocampal PS was demonstrated in senescence accelerated mice model, which has a short life span, memory and learning alterations, and increased amounts of hippocampal Aβ plaques. In addition, DHA content of PS and PE are 12 and 14% reduced in the human AD’s cortex respectively, and also it has been found less amount of PE subspecies 22:6 n-3 (DHA) in the cortex of 9-month-old AD mice (Cunnane et al., 2012). In general, a reduction of PE, PE-derived PUFAs in the brain (Ginsberg et al., 1993; Conquer et al., 2000; Dorninger et al., 2015) and in plasma (Conquer et al., 2000) has been associated to AD. Therefore, our results are in agreement with the previous findings and suggest that altered PS and PE metabolism in the hippocampus could be implied in the cognitive impairment described in AD by affecting the signaling pathways modulated by PUFAs, such as DHA and AA.

PE accounts for approximately 25% of mammalian PLs and is significantly enriched in the brain, where the PE content is 45% of the total PLs. PE has been recently involved in the positive regulation of autophagy and longevity (Rockenfeller et al., 2015). PEs and/or PE-derived (LPE and ePE) are essentials in the cell function (Vance and Tasseva, 2013), LPE can be generated from PE via a phospholipase A-type reaction (Vance and Tasseva, 2013). Currently, the physiological significance of LPE in the brain is unknown. However, in non-mammalian species, it has been attributed to certain functions. For example, in the housefly, LPE has antifungal and antibacterial activity (Meylaers et al., 2004), and in the mushroom *Grifola frondosa*, it stimulates mitogen-activated protein kinase (MAPK) signaling. Furthermore, was reported that LPE induced neuronal differentiation of PC12 cells (Nishina et al., 2006). And recently it has been reported that LPE has a direct relationship with the calcium influx (Lee et al., 2015), which is close related to cell death (Bezprozvanny and Mattson, 2008). Interestingly, our data indicated increased LPE levels in the hippocampus of AD animals (Scr6 m and Scr12 m), specifically, the AA (20:4), the DHA (22:6) and the oleic acid (18:1) composition. Surprisingly, BACE1 silencing for 6 and 12 months reestablished the levels of these FAs to the control levels, which suggested that LPE is accumulated in AD. In addition, The PLS-DA analysis of the phospholipid species, also suggested that, the shBACE1miR treatment generated a displacement of lipid prolife from AD group to control group profile. According to PLS-DA, such displacement is explained in an important part by LPE (16:1, 18:1, 20:4), meaning a significant role of BACE1 silencing in neuroprotection.

Although, DHA is widely reported as being beneficial for cells, the excess levels likely trigger a negative effect on the membrane balance because the DHA content in the plasma membrane of T27A cells may be detrimental. This prediction was confirmed by observations of the reduced viability of cells incubated with 22:6/18:0 (Zerouga et al., 1996). In contrast, the key enzyme involved in the turnover and degradation of LPE, phospholipase A_2_ (PLA_2_), is markedly increased in brains from AD patients (Farooqui, 2010) and associated to AA production in AD (Sanchez-Mejia and Mucke, 2010). Also, PLA_2_ activation has been linked to inflammation process in a genetically model of neurodegeneration, since it showed that specific lysophospholipids were responsible for microglial activation and the cytosolic PLA2 (cPLA2) inhibition had neuroprotective effect (Lee et al., 2011; Sundaram et al., 2012). According to our results, BACE1 silencing down-regulated the composition of AA in LPE at 6 and 12 months post-treatment, and decreased the phosphorylation state of cPLA_2,_ which could be involved in the cell membrane homeostasis of treated AD mice at 12 months post-injection, with the consequent down-regulation of AA levels and COX2 pro-inflammatory pathway in the same time-line (**Figures 7C,D**), suggesting a more effective anti-inflammatory regulation by the long-term BACE1’s silencing. Which are in concordance with our recent data were BACE1 silencing reduced not only β-amyloid and CTF, but also reduced MAPK activity and phosphorylated soluble tau (Piedrahita et al., 2016). MAPK regulates cPLA2 activity down-stream under lipid peroxidation condition (Lee et al., 2011). Also, cPLA2 activity has been involved in the hyperphosphorylation of tau during neuroinflammation (Sundaram et al., 2012). AA through COX2 activity is the precursor of pro-inflammatory eicosanoids, prostaglandin E2 and leukotriene B4 and also increases the production of interleukin-1, tumor necrosis factor-α and IL-6 (SanGiovanni and Chew, 2005; Freund-Levi et al., 2014).

On the other side, also it has been reported that 22:6 FA suppresses the production of AA-derived eicosanoids and thus exerts anti-inflammatory and immunosuppressive effects (Calder, 2012). Decreased levels of 22:6 trigger oxidative stress, which is known as a primary factor in the pathogenesis of AD because of the high-metabolic rate of the brain, which makes it particularly susceptible to ROS (Migliore et al., 2005). Also, oxidative stress is related to the progressive degradation of brain PLs in AD because they are rich in readily oxidizable AAs and DHAs (SanGiovanni and Chew, 2005; Calder, 2012). However, our data suggest a membrane destabilization process showing an up-regulation of DHA 22:6 in the LPE composition, maybe contributing to the pathogenesis of AD. Interestingly our data suggest that the BACE1 knock-down reverse this pathogenic environment, generating homeostasis on FA composition of LPE, and reducing pro-inflammatory pathway cPLA2/AA/COX-2.

On the complementary side, a deficiency in brain ePE, which constitutes up to 70% of the total plasmalogens, has been traditionally associated with AD development, and low levels have been detected in the brain (Braverman and Moser, 2012), in the cerebrospinal fluid (Brites et al., 2009; Andrade-Moraes et al., 2013) and in the serum of patients (Lessig and Fuchs, 2009); supporting the importance of those ones to control the membrane instability during oxidative stress in AD. According to our results, the reduction in ePE levels resulted from a significant decrease in the polyunsaturated 22:6 (DHA) and 18:0 (oleic acid) FAs. Because plasmalogens function as “scavengers” of ROS in biological membranes, low levels of ePE are associated with reduced cell viability because of the cell loss of antioxidant capacity. Decreased concentrations of ePE may be correlated with peroxisomal alterations because the enzymes required for its biosynthesis are found in these organelles (Lizard et al., 2012). Several studies have reported alterations in peroxisomes isolated from AD brains and reduced biosynthesis of plasmalogens composed of DHA (Kou et al., 2011; Fanelli et al., 2013). Furthermore, the main ePE degradation enzyme is PLA_2_, which is significantly increased in AD. Interestingly, in this work, we demonstrated that BACE1 silencing restored the amount of ePE in the hippocampus of 3xTg-AD mice to the control levels, suggesting that BACE1 silencing may play a role in the regulation of PLA_2_ activity restoring the homeostasis of this plasmalogen and allowing it to reach effective concentrations for antioxidant activity.

In summary, our study suggests that AD is associated with a general disruption of membrane properties and a plasmalogens composition disbalance. The length and saturation number are important determinants of many membrane characteristics including membrane, fluidity, thickness and the local curvature, even molecular packing, which in turn regulate the activities of the enzyme membrane. Intramembrane proteolysis is likely to be exquisitely regulated by the thickness of the membrane (Hong, 2015), which could have implications for β-secretase-mediated cleavage of APP. However, this work suggests by the first time that BACE1 is involved in FA composition of PE-derivates (LPE and ePE), and that the shBACE1miR treatment in 3xTg-AD mice restores the levels of lipid subspecies that are altered by the disease in the animal model, preventing pro-inflammatory signaling, which could be associated the reduction of tauopathy and with the learning and memory improvement. However, further research is necessary to establish whether there is a direct or indirect correlation between the BACE1 levels and the metabolic pathways of PE, LPE, and ePE and to explain how BACE1 silencing favors the appearance of the PUFAs involved in neuroprotection or if the reduction of BACE1 attenuated cytotoxic βA production for restoring cellular function including lipid metabolism, anti-inflammatory pathway and cognitive function, and to verify which would be the analog modulation of the phospholipid profile and composition in the human AD pathogenesis.

## Author Contributions

JV-O design, acquisition of data, analyses and interpretation of data, write the manuscript; AB-O design, acquisition of data, analysis and interpretation of data; DP design, acquisition of data, analysis and interpretation of data; CV-R analysis data, critical revision; JA-L analysis data, critical revision; GC-G design, acquisition of data, analysis and interpretation of data, write the manuscript.

## Conflict of Interest Statement

The authors declare that the research was conducted in the absence of any commercial or financial relationships that could be construed as a potential conflict of interest.
