# Supplementary figures and images for: BACE1 RNAi Restores the Composition of Phosphatidylethanolamine-Derivates Related to Memory Improvement in Aged 3xTg-AD Mice
Source: Front Cell Neurosci. 2016 Nov 11;10:260. doi: 10.3389/fncel.2016.00260 (PMC5105502; doi:10.3389/fncel.2016.00260)

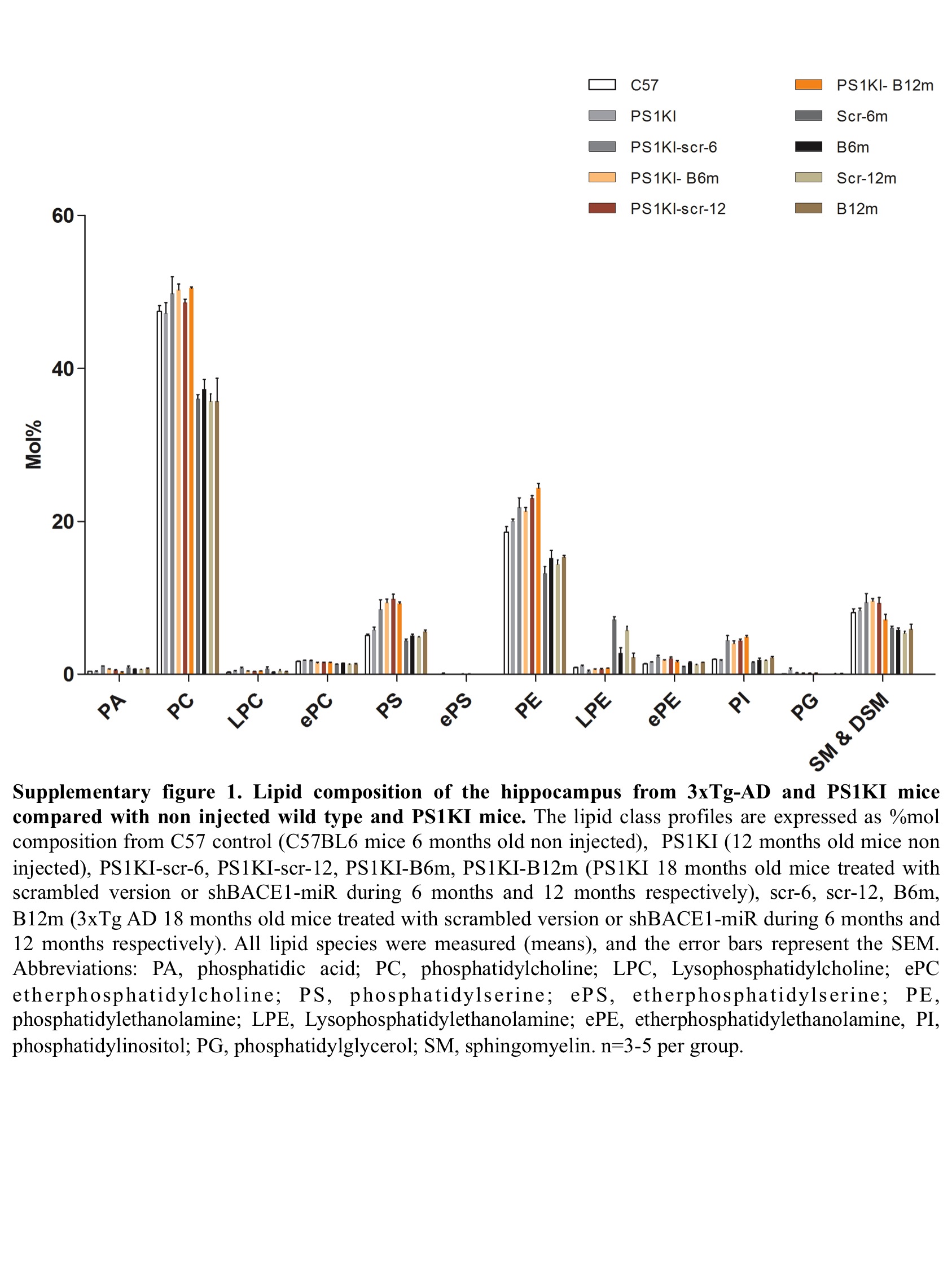

Supplement: Supplementary file 1 [file Image_1.jpg]
